# A different surgical approach for cardiophrenic lymph node resection in advanced ovarian cancer

**DOI:** 10.3332/ecancer.2017.780

**Published:** 2017-11-16

**Authors:** Lucas Minig, Miguel Arraras, Cristina Zorrero, Pedro Martinez, Manuel Patron, Juan Carlos Peñalver

**Affiliations:** 1 Gynecology Department, Instituto Valenciano de Oncología (IVO), Valencia 46009, Spain; 2 Thoracic Surgery, Instituto Valenciano de Oncología (IVO), Valencia 46009, Spain

**Keywords:** advanced stage, ovarian cancer, cardiophrenic lymph node, surgical technique, subxiphoid approach

## Abstract

**Objective:**

To describe the surgical technique of a **subxiphoid approach** to remove cardiophrenic lymph nodes in women with advanced ovarian cancer.

**Materials and methods:**

The first step is to dissect and separate the anterior insertions of the diaphragm at the xiphoid appendix. Thus, the parietal peritoneum and the upper fibres of the transversus abdominis muscle are incised. Then, the anteroinferior mediastinum is identified and dissected. Diaphragmatic deinsertion may be extended 5–7 cm laterally to the xiphoid appendix following the inferior costal margin according with the localization of the enlarged lymph nodes. Thus, the dissection of the anterolateral cardiophrenic space allows the identification of both pleura. In addition, the vertical dissection of the anterior cardiophrenic space allows the removal of enlarged lymph nodes. However, it is important to bear in mind at this time that the unintentional opening of the pleura is possible. To reduce this risk, a careful dissection of the anterior cardiophrenic fat tissue is essential. Moreover, a careful dissection will avoid damage at the left phrenic nerve as well as the left pericardiophrenic artery and vein. After removing the cardiophrenic fat tissue, the diaphragm is sutured at the lower costal margin by using separated stiches of absorbable 2-0 suture.

**Conclusion:**

The subxiphoid approach to resect cardiophrenic lymph nodes is a feasible surgical technique. In addition, it reduces the possibility of opening the pleural cavity, while avoiding a diaphragmatic incision, in comparison with the standard trans-diaphragmatic surgical approach.

## Introduction

Ovarian cancer is the sixth most common cancer among women in Europe and the most aggressive gynecological malignancy. This is mainly due to the fact that over 80% of cases are diagnosed at an advanced stage of disease. International guidelines currently recommend achieving a complete tumour resection at the time of surgical cytoreduction [1, 2]. In order to achieve this goal, however, several complex procedures are being incorporated as part of the standard surgical procedures [3, 4]. In this regard, aimed at obtaining a complete tumour resection, the removal of the cardiophrenic lymph node has been recently proposed [5].

The first study proposed the removal of cardiophrenic nodes by video-assisted thoracic surgery (VATS) [5]. More recently, a trans-diaphragmatic resection of cardiophrenic lymph nodes has been described [3, 6–9]. However, postoperative complications such as pleural effusion, pneumothorax, respiratory failure, or pneumonia have been associated with this surgical approach [3]. Therefore, the subxiphoid surgical technique to remove cardiophrenic lymph nodes in women with advanced ovarian cancer is described in this article.

## Materials and methods

A 67-year-old woman, performance status ‘0’ without co-morbidities, was received at the Department of Gynecology, Instituto Valenciano de Oncología, Valencia, Spain. The level of CA-125 was 1265 UI/mL; CEA: 1.2 ng/mL; CA-19.9: 12 UI/mL. The CT scan showed a 12-cm suspected ovarian mass, four-quadrant ascites, peritoneal carcinomatosis and a 1.2-cm cardiophrenic lymph node at the midline. No pleural effusion, nor lung or intra-hepatic metastases were observed. The patient underwent a complete primary debulking surgery, where a Type II radical oophorectomy with immediate bowel anastomosis, right diaphragm and Morrison’s pouch peritonectomy and splenectomy were performed. An enlarged cardiophrenic lymph node was removed as well, following the surgical technique described below.

### Surgical technique

**Figure 1.**
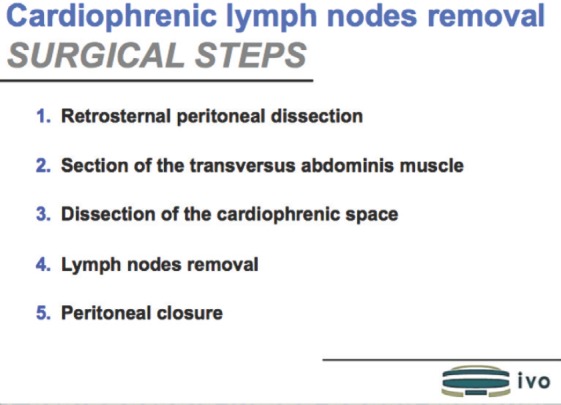
Surgical steps.

**Figure 2.**
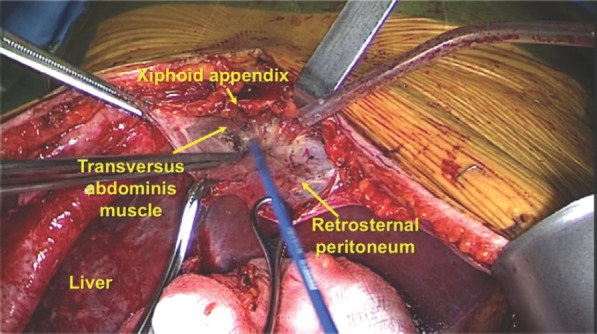
Anatomical structures before entering into the anterior cardiphrenic space by a subxiphoid approach. To view this video, click here https://ecancer.org/journal/11/780-a-different-surgical-approach-for-cardiophrenic-lymph-node-resection-in-advanced-ovarian-cancer.php.

The anterior cardiophrenic space is located between the sternum and the heart, and above the diaphragm. It contains fat tissue with lymph nodes, which drain from both diaphragms. The first step is to dissect and separate the anterior insertions of the diaphragm at the xiphoid appendix. Thus, the parietal peritoneum and the upper fibres of the transversus abdominis muscle are incised until the diaphragm is identified (Figure 1). Then, the anteroinferior mediastinum is identified and dissected. At this point, the pericardial sac is also identified. Diaphragmatic deinsertion may be extended 5–7 cm laterally to the xiphoid appendix, following the inferior costal margin according with the localisation of the enlarged lymph nodes (Figure 2). Thus, the dissection of the anterolateral cardiophrenic space allows the identification of both pleura. In addition, the vertical dissection of the anterior cardiophrenic space allows the removal of enlarged lymph nodes. However, it is important to take in mind, at this time, that the unintentional opening of the pleura is possible. To reduce this risk, a careful dissection of the anterior cardiophrenic fat tissue is essential. Moreover, a careful dissection will avoid damages at the left phrenic nerve as well as the left pericardiophrenic artery and vein. The fourth surgical step is the removal of the cardiophrenic fat tissue with lymph nodes. Finally, the diaphragm is sutured at the lower costal margin by using separated stiches of absorbable 2-0 suture.

## Results

The surgical time was 380 min, with an estimated blood loss of 1200 ml. Two packs of red blood cells were required intra-operatively. The cardiophrenic node removal lasted 17 min from step 1 to step 5, with an unappreciable blood loss. After two days of recovery in the intensive care unit, she completed the postoperative course at the general ward. An X-ray performed during the first postoperative day revealed neither pneumothorax nor pleural effusion. She was discharged ten days after surgery without major complications. The pathological diagnosis revealed a high-grade serous epithelial ovarian cancer FIGO stage IVB due to a positive cardiophrenic lymph node. She initiated adjuvant chemotherapy based on intravenous carboplatin/paclitaxel/bevacizumab 34 days after surgery.

## Discussion

The cardiophrenic angle lymph nodes include two different groups of nodes: the anterior pre-pericardiac and the middle latero-pericardiac ones. The afferent lymphatics of cardiophrenic lymph nodes drain areas from the diaphragm, liver, pleura and anterior abdominal wall, and they empty into the internal mammary chain [10].

There are some controversies regarding the minimum size of lymph nodes to be resected at debulking surgery for ovarian cancer. A recent study evaluated 31 patients with cardiophrenic nodes resection as part of cytoreduction for advanced ovarian cancer. Histological analysis of the lymph nodes observed metastasis in 19 out of 31 patients (61.3%). Analysis of the ROC curves observed that the short axis 7 mm cutoff showed the area under the largest curve with a sensitivity of 61% and a specificity of 83% [6]. Another study of 22 patients with excised cardiophrenic ganglia observed metastases in 21 of them, and all had a size of at least 8 mm at the short axis [7].

To date, seven studies have demonstrated the feasibility and safety of excising cardiophrenic lymph nodes in the context of ovarian cancer cytoreduction [3, 5–9, 11]. Even though the first studies described the surgical approach by VATS [5, 11], the more recent studies described a trans-diaphragmatic approach [3, 6–9]. In the latter approach, a liver mobilisation and diaphragmatic incision is required, even in some circumstances after diaphragmatic peritonectomy when the pleural space was not opened. Therefore, postoperative complications such as pleural effusion, pneumothorax, respiratory failure or pneumonia have been reported to be associated with this surgical approach [3]. Thus, the surgical approach we describe avoids the necessity to open the diaphragm as well as the pleural space. However, it is important to highlight that a careful dissection of the cardiophrenic fat tissue is required to avoid unintentional pleural opening. Therefore, this approach should be considered when the opening of the diaphragm muscle is avoided during diaphragm peritonectomy.

## Conclusion

Utilising the subxiphoid approach to remove cardiophrenic lymph nodes is a feasible surgical technique. In addition, this approach reduces the possibility of opening the pleural cavity, while avoiding a diaphragmatic incision, in comparison with the standard trans-diaphragmatic surgical approach.

## Conflict of interest statement

The authors declare that there are no conflicts of interest.

## References

[1] Querleu D (2016). European Society of Gynaecologic Oncology Quality Indicators for Advanced Ovarian Cancer Surgery. Int J Gynecol Cancer.

[2] Wright AA (2016). Neoadjuvant chemotherapy for newly diagnosed, advanced ovarian cancer: Society of Gynecologic Oncology and American Society of Clinical Oncology Clinical Practice Guideline. Gynecol Oncol.

[3] LaFargue CJ (2016). Short-term morbidity in transdiaphragmatic cardiophrenic lymph node resection for advanced stage gynecologic cancer. Gynecol Oncol Rep.

[4] Chi DS (2009). Improved progression-free and overall survival in advanced ovarian cancer as a result of a change in surgical paradigm. Gynecol Oncol.

[5] Lim MC (2009). Pathological diagnosis and cytoreduction of cardiophrenic lymph node and pleural metastasis in ovarian cancer patients using video-assisted thoracic surgery. Ann Surg Oncol.

[6] Kim TH (2016). Preoperative prediction of cardiophrenic lymph node metastasis in advanced ovarian cancer using computed tomography. Ann Surg Oncol.

[7] Garbi A (2017). Feasibility of transabdominal cardiophrenic lymphnode dissection in advanced ovarian cancer: initial experience at a tertiary center. Int J Gynecol Cancer.

[8] Prader S (2016). Surgical management of cardiophrenic lymph nodes in patients with advanced ovarian cancer. Gynecol Oncol.

[9] Cowan RA (2017). Feasibility, safety and clinical outcomes of cardiophrenic lymph node resection in advanced ovarian cancer. Gynecol Oncol.

[10] Ragusa M (2011). Isolated cardiophrenic angle node metastasis from ovarian primary. Report of two cases. J Cardiothorac Surg.

[11] Yoo HJ (2013). Transabdominal cardiophrenic lymph node dissection (CPLND) via incised diaphragm replace conventional video-assisted thoracic surgery for cytoreductive surgery in advanced ovarian cancer. Gynecol Oncol.

